# Comparative Phenotypic and Functional Analyses of the Effects of IL-10 or TGF-β on Porcine Macrophages

**DOI:** 10.3390/ani11041098

**Published:** 2021-04-12

**Authors:** Tania Carta, Elisabetta Razzuoli, Floriana Fruscione, Susanna Zinellu, Dionigia Meloni, Antonio Anfossi, Bernardo Chessa, Silvia Dei Giudici, Simon P. Graham, Annalisa Oggiano, Giulia Franzoni

**Affiliations:** 1 Department of Animal Health, Istituto Zooprofilattico Sperimentale della Sardegna, 07100 Sassari, Italy; tcarta@uniss.it (T.C.); Susanna.Zinellu@izs-sardegna.it (S.Z.); dionigia.meloni@izs-sardegna.it (D.M.); Silvia.DeiGiudici@izs-sardegna.it (S.D.G.); 2 School of Veterinary Medicine, University of Sassari, 07100 Sassari, Italy; aanfossi@uniss.it (A.A.); bchessa@uniss.it (B.C.); 3 National Reference Center of Veterinary and Comparative Oncology (CEROVEC), Istituto Zooprofilattico Sperimentale del Piemonte, Liguria e Valle d’Aosta, Piazza Borgo Pila 39/24, 16129 Genoa, Italy; elisabetta.razzuoli@izsto.it (E.R.); floriana.fruscione@izsto.it (F.F.); 4 Porcine Reproductive and Respiratory Syndrome (PRRS) Immunology, The Pirbright Institute, Ash Road, Pirbright, Woking GU24 ONF, UK; simon.graham@pirbright.ac.uk

**Keywords:** pig, macrophages, IL-10, TGF-β, cytokines, surface markers, TLR4 agonist, TLR2 agonist

## Abstract

**Simple Summary:**

Macrophages play a central role in innate immune response to both infectious and non-infectious stressors. They respond to different agonists modifying their phenotype and functions. In humans and mice, the regulatory cytokines IL-10 or TGF-β are both known to drive macrophage polarization into an anti-inflammatory phenotype, referred to as M2c. However, the immune systems of animal species each have their own peculiarities and the M2c subsets has never been investigated in pigs. A deep knowledge of the porcine immune system is required to design vaccines or control strategies against pathogens, which are a major constraint to pork production. Due to anatomical, physiological, and immunological similarities, swine are attracting increasing attention as a model for human diseases. To better characterize porcine macrophages, we evaluated the effects of IL-10 or TGF-β on the phenotype and function of monocyte-derived macrophages. Both cytokines downregulated the expression of MHC II DR and CD14. IL-10, but not TGF-β, statistically significantly reduced the ability of macrophages to respond to Toll-like receptor 2 (TLR2) or TLR4 agonists. Whilst these data suggest differentiation to an M2c-like immunosuppressive phenotype, the responses, and differences between IL-10 and TGF-β also reveals species-specific differences.

**Abstract:**

Macrophages are phagocytic cells involved in maintaining tissue homeostasis and defense against pathogens. Macrophages may be polarized into different functionally specialized subsets. M2c macrophages arise following stimulation with IL-10 or TGF-β and mediate anti-inflammatory and tissue repair functions. M2c macrophages remain poorly characterized in the pig, thus we investigated the impact of these regulatory cytokines on porcine monocyte-derived macrophages (moMΦ). The phenotype and functionality of these cells was characterized though confocal microscopy, flow cytometry, ELISA, and RT-qPCR. Both cytokines induced CD14 and MHC II DR down-regulation and reduced IL-6, TNF-α, and CD14 expression, suggestive of an anti-inflammatory phenotype. Interestingly, neither IL-10 or TGF-β were able to trigger IL-10 induction or release by moMΦ. Differences between these cytokines were observed: stimulation with IL-10, but not TGF-β, induced up-regulation of both CD16 and CD163 on moMΦ. In addition, IL-10 down-regulated expression of IL-1β and IL-12p40 4h post-stimulation and induced a stronger impairment of moMΦ ability to respond to either TLR2 or TLR4 agonists. Overall, our results provide an overview of porcine macrophage polarization by two immunosuppressive cytokines, revealing differences between IL-10 and TGF-β, and reporting some peculiarity of swine, which should be considered in translational studies.

## 1. Introduction

Macrophages (Mφ) are phagocytic cells, which play a central role in tissue homeostasis, by clearing cell debris, dying cells, and repairing tissues after inflammation [1,2]. They are crucial in immune response to pathogens, being able to detect foreign molecules through an array of sensing molecules, named pattern recognition receptors (PRRs) [3,4]. Macrophages also contribute to the initiation of acquired immune responses by processing and presenting antigens to naïve T lymphocytes [3]. Mφ are a heterogenous population, with subsets in different anatomic locations presenting discrete functional characteristics (reviewed by [2]). Mφ may also be differentially activated, resulting in their polarization into different functionally specialized subsets, referred to as ‘classically’ (M1) and ‘alternatively’ (M2) activated Mφ [5]. Classical activation by interferon-gamma (IFN-γ) and lipopolysaccharide (LPS) produces M1 Mφ with an increased microbicidal or tumoricidal capacity to better mediate defense to intracellular pathogens. Alternative activation induces M2 Mφ, which are primarily associated with mechanisms of wound repair and immunosuppression [5]. M2 Mφ were recently classified into three further subsets: M2a (activated with IL-4 or IL-13), M2b (activated by exposure of immune complexes in combination with IL-1β or LPS) and M2c (stimulated with IL-10, TGF-β or glucocorticoids) [4]. Overall, macrophages present remarkable malleability, with constant variations in their phenotype and functionality in response to subtle and continuously changing milieu of signals [1], thus, it was suggested to adopt a different nomenclature for their classification, linked to the activator/s used, e.g., M(IL-4), M(IFN-γ), M(IL-10), M(LPS), M(Ig) [6]. Despite the crucial importance of polarization on Mφ phenotype and functionality, few studies have analyzed Mφ polarization in pigs [7,8,9].

Pork production represents more than one-third of global meat production, and infectious diseases represent its main constraint, being responsible for major economic losses [10]. Knowledge of the porcine immune system is mandatory to understand how pigs respond to pathogens, and to rationally design vaccines or other biological interventions to control infectious diseases [11]. In addition, swine are attracting increasing attention as a biomedical model due to their anatomical, physiological, and immunological similarities to humans [12,13,14,15]. For example, pigs are used in pre-clinical toxicologic testing of pharmaceuticals [13], pre-clinical evaluation of vaccine candidates and therapeutics [16,17], and in nanomedicine studies [18,19]. A recent comparison of the porcine and human immune system showed similarities in over 80% of analyzed parameters, whereas mice and humans shared only about 10% [15]. For example, pigs and humans both have tonsils, which are absent in rodents, and the immunology of pig skin makes it an ideal model for human skin [14,15]. In addition, swine are monogastric and omnivorous, thus are more suitable than some other species to study intestinal immunology [15].

Current knowledge of the porcine immune system presents several gaps and few studies have focused on Mφs in different polarized states. As described in humans, generation of porcine M1 resulted in release of pro-inflammatory cytokines (IL-1β, IL-6, IL-8, TNF-α), and up-regulation of the activation marker CD25 [7,8,9]. In addition, classical activation of porcine macrophages resulted in up-regulation of molecules involved in antigen presentation, such as MHC class I and II and co-stimulatory molecules CD80/CD86 and CD40 [7,8,9]. On the contrary, activation of porcine Mφ with IL-4 induced expression of the M2a key effector arginase-1 (Arg-1) (an enzyme involved in catalyzing the hydrolysis of arginine to ornithine) and up-regulation of the surface marker CD203a (ectonucleotide pyrophosphatase/phosphodiesterase 1) [7,9]. We also reported that Mφ in antithetic polarized status responded differently to myelotropic viruses such as porcine reproductive and respiratory syndrome virus (PRRSV) and African swine fever virus (ASFV) [7,20,21]. Polarization of porcine macrophages with IFN-γ and LPS (classical activation), but not IL-4, resulted in higher resistance to infection with either PRRSV or ASFV [7,20,21].

However, no studies to date have deeply analyzed other M2 subtypes, like M2c, in the pig. The aim of this work was therefore to evaluate the effects of IL-10 and TGF-β (two immunosuppressive cytokines [22]) on Mφ phenotype and functionality though an integrative analytical approach, using confocal microscopy, flow cytometry, multiplex cytokine ELISA, and RT-qPCR.

## 2. Materials and Methods

### 2.1. Ethical Statement

Eight healthy pigs (*Sus scrofa domesticus*) were utilized for blood sampling. Pigs were cross-bread, 6–24 months old, and were kept at the Experiment Station of the Istituto Zooprofilattico Sperimentale (IZS) of Sardinia. Blood sampling was approved by the local ethics committee, in accordance with the Guide of Use of Laboratory Animals issued by the Italian Ministry of Health.

### 2.2. Generation of Porcine Monocyte-Derived Macrophages and Activation

Mφ cultures were obtained from blood leukocytes, as previously described [21]. Leukocytes were cultured for 7 days in RPMI-1640 supplemented with 10% fetal bovine serum (FBS), 100 U/mL streptomycin, and 100 μg/mL penicillin (complete RPMI, cRPMI), and with 50 ng/mL of recombinant human M-CSF (hM-CSF) (Thermo Fisher Scientific, Waltham, MA, USA), using Petri dishes, as previously described [21]. MoMΦ were then harvested, washed, re-suspended in cRPMI and seeded in 12-well plates (Greiner CELLSTAR, Sigma-Aldrich, Saint Louis, MO, USA) (8–10 × 10^5^ live cells per well) or 8-well chamber slides (Nunc Lab-Tek chamber slide system, Sigma-Aldrich) (1 × 10^5^ live cells per well). Cells were incubated at 37 °C 5% CO_2_ for further 24 h before stimulation. moMΦ were left untreated or stimulated with recombinant porcine IL-10 or TGF-β: culture medium was replaced with fresh cRPMI containing either IL-10 (20 ng/mL) or TGF-β (20 ng/mL) (both R&D Systems, Minneapolis, MN, USA). In defined experiments, culture medium was instead replaced with fresh cRPMI containing LPS (lipopolysaccharide from *Escherichia coli* 0111:B4; Sigma-Aldrich) (1 μg/mL) or recombinant porcine IL-4 (20 ng/mL) (R&D Systems), which were used as positive controls.

### 2.3. Impact of IL-10 or TGF-β Activation on moMΦ Phenotype

MoMΦ were cultured in 12 well plates (to assess surface marker expression by flow cytometry) or 8-well chamber slides (for confocal microscopy). Then, 24 h post-activation with IL-10 or TGF-β, moMΦ phenotype was assessed by confocal microscopy and flow cytometry.

For confocal microscopy, moMΦ were fixed using 4% paraformaldehyde and then labelled with Alexa Fluor 488 conjugated phalloidin and Hoechst 33342 (both Molecular Probes, Thermo Fisher Scientific, Rockford, USA) to visualize actin cytoskeleton or nuclei, respectively [20]. Images were acquired on a format of 1024 × 1024 pixels, using a Leica SP5 Confocal Microscope (Leica Microsystem, Wetzlar, Germany) equipped with a 40X Plan-Apo 1.25 NA oil immersion objective. Images were then processed with LAS AF Lite software (Leica Microsystem) as previously described [20].

Flow cytometry was carried out as previously described, with slight modifications [21]. In brief, moMΦ were harvested using PBS supplemented with 10 mM EDTA and then transferred into 5 mL round bottom tubes (Corning). First, cells were stained with Zombie Aqua viability dye (BioLegend, San Diego, CA, USA) for 30 min at room temperature (RT). Then, moMΦ were washed with PBS supplemented with 0.5% bovine serum albumin (BSA) and then stained with the following murine monoclonal antibodies: anti-human CD14-PerCP-Cy5.5 (clone Tuk4; Miltenyi Biotec, Bergisch Gladbach, Germany), anti-porcine MHC II DR-FITC (clone 2E9/13, Bio-Rad Antibodies, Kidlington, United Kingdom), CD16-PE (clone G7, Thermo Scientific Pierce, Rockford, IL, USA), CD163-PE (clone 2A10/11, Bio-Rad Antibodies), CD169-FITC (clone 3B11/11, Bio-Rad Antibodies), and CD25 (clone K231.3B2, Bio-Rad Antibodies). CD25 expression was visualized by subsequent staining with BV421 rat anti-mouse IgG1 (clone A85-1, BD Horizon BD Biosciences, Franklin Lakes, NJ, USA). Incubations with either primary or secondary antibodies were carried out for 15 min at 4 °C. After washing with PBS supplemented with 2% FBS, moMΦ were resuspended in PBS supplemented with 2 mM EDTA and were analyzed with a FACS Celesta (BD Biosciences). A total 5000 live moMΦ were acquired and analysis of data was performed using BD FACS Diva Software (BD Biosciences): first doublets were excluded, then moMΦ were gated according to FSC and SSC values, dead cells excluded according to viability staining, and finally staining for surface markers were assessed.

### 2.4. Induction of Cytokine Release in Response to IL-10 or TGF-β Activation

MoMΦ were left untreated or stimulated with IL-10 or TGF-β and 24 h later cytokine levels in culture supernatants were evaluated. LPS was used as a positive control. Culture supernatants were harvested, cleared of debris by centrifugation (2000× *g* for 3 min) and stored at −80 °C until analyzed. Levels of IL-1α, IL-1β, IL-6, IL-10, IL-12, and TNF-α were determined using Porcine Cytokine/Chemokine Magnetic Bead Panel Multiplex assay (Merck Millipore, Darmstadt, Germany) and a Bioplex MAGPIX Multiplex Reader (Bio-Rad, Hercules, CA, USA), following the manufacturers’ instructions, as previously described [20]. 

### 2.5. Impact of IL-10 or TGF-β Activation on Different Cytokines, and CD14, TLR4, Arginase-1 Gene Expression

MoMΦ were left untreated or stimulated with IL-10 or TGF-β; at selected time points (0, 4, 8, 24 h) gene expression of five different cytokine (IL-1β, IL-6, IL-10, IL-12p40, TNF-α), CD14, TLR4, Arg-1 was determined. For Arg-1 gene expression, stimulation with IL-4 was used as a positive control. First, total RNA was extracted from moMΦ using the RNeasy Mini Kit (QIAGEN, Hilden, Germany) and finally eluted in 50 µL of ultrapure RNase-free water. Then, 100 ng of purified RNA was used as template for cDNA synthesis, as previously described [23]. Gene expression was evaluated by RT- qPCR, using previously reported primer sets: Il-1β, IL-6, IL-18, TNF-α, CD14, TLR4 [23]; IL-12p40 [24]; IL-10 [25]; Arg-1 [9]. RT-qPCR of cytokines, CD14, and TLR4 were performed at the CEROVEC (Genoa, Italy); EVA Green Real-Time PCR amplification was performed in a CFX96™ Real-Time System after the reverse transcription step, using glyceraldehyde 3-phosphate dehydrogenase (GAPDH) as reference gene, as we previously described [23]. RT-qPCR of Arg-1 were instead carried out at the IZS of Sardinia (Sassari, Italy). For this gene, beta-actin (ACTB) was instead used as reference, using forward (5-CTCGATCATGAAGTGCGACGT-3) and reverse (5-GTGATCTCCTTCTGCATCCTGTC-3) primers targeting a 114 base pair region, and the probe 5-TET-ATCAGGAAGGACCTCTACGCCAACACGG-BHQ1-3 [26]. For Arg-1, PCR amplifications were performed in a final volume of 10 µL, containing 0.6 µM of forward and reverse primers and 0.3 µM of probe. Real time PCR was performed in a 7500 Fast Real Time PCR System (Thermo Fisher) with the following protocol: initial denaturation 95 °C for 10 min, followed by 45 cycles of 95 °C for 10 s, 60 °C for 30 s. For all tested genes, five independent experiments were carried out, using different blood donor animals. In each sample, the relative expression of the test genes was calculated using the widely adopted 2−ΔΔCt method: Ct is acronym for cycle of threshold, ΔCt = Ct (target gene) − Ct (reference gene), and ΔΔCt = ΔCt (stimulated samples) − ΔCt (un-treated sample, moMΦ) [21,23].

### 2.6. Impact of IL-10 or TGF-β Activation on moMΦ Ability to Respond to Stimulation with TLR4 or TLR2 Agonist

MoMΦ were left untreated or stimulated with IL-10 or TGF-β, as above described. Then, 24 h later, media was removed and replaced with cRPMI supplemented with either a TLR2 agonist (100 ng/mL) or a TLR4 agonist (1 μg/mL), and after 24 h supernatants were harvested, cleared of debris by centrifugation (2000× *g* for 3 min) and stored at −80 °C until determination of cytokine levels. Levels of IL-1α, IL-1β, IL-6, IL-12, TNF-α were determined using Porcine Cytokine/Chemokine Magnetic Bead Panel Multiplex assay, as described above. A S-[2–bis(palmitoyl)-propyl]cysteine (Pam2Cys) lipopeptide was used as TLR2 agonist; it was synthesized based on the 14 amino acids following the cysteine immediately downstream the signal peptide of a *Mycoplasma agalactiae* lipoprotein (P48: CGDKYFKETEVDGV) (Espikam, Prato, Italy) [27]. LPS from *E. coli* 0111:B4 (Sigma-Aldrich) was used as a TLR4 agonist.

### 2.7. Statistical Analysis

In vitro experiments were carried out in technical duplicate and repeated with three (confocal images, multiplex ELISA) or four (flow cytometry) or five (RT-qPCR) different pigs. Baseline data distribution was determined based on Shapiro–Wilk test using R-software, version 3.6.2 (R-Foundation for Statistical Computing, Vienna, Austria), and then data were graphically and statistically analyzed with GraphPad Prism 8.01 (GraphPad Software Inc, La Jolla, USA). Data were presented as mean and standard error of the mean (SEM) and were analyzed with the parametric one-way ANOVA followed by Dunnett’s multiple comparison test or the non-parametric Kruskal–Wallis test followed by a Dunn’s multiple comparison test. A *p* value of less than 0.05 was considered statistically significant. Statistically significant differences were displayed in the figures using the following symbols: *p* < 0.05 (*), *p* < 0.01 (**), *p* < 0.001 (***).

## 3. Results

### 3.1. Impact of IL-10 or TGF-β on Porcine moMΦ Phenotype

**Figure 1:**
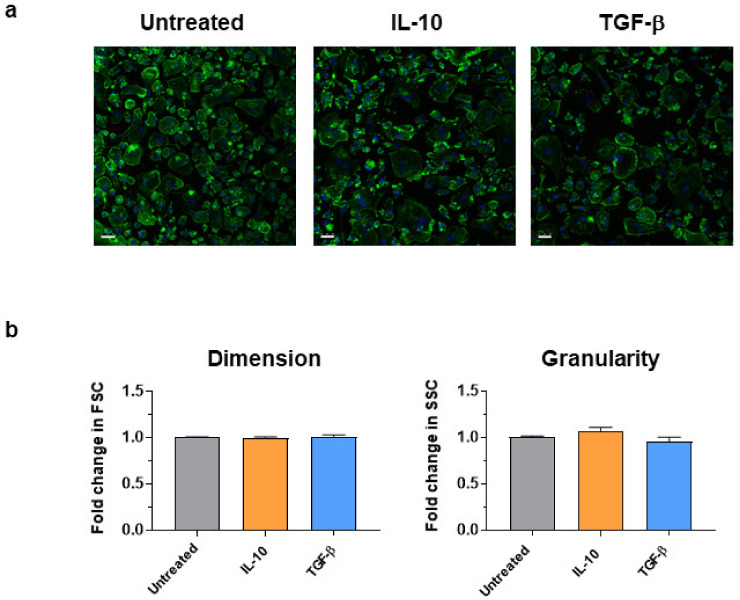
Morphological analysis of moMΦ stimulated with IL-10 or TGF–β. 24 h post-stimulation with either IL-10 or TGF-β, morphology were assessed with confocal microscopy and flow cytometry. (**a**) Confocal microscopy observation after nuclei staining with Hoechst 33342 (blue) and cytoskeleton with Alexa Fluor 488-conjugated phalloidin (green), with magnification 40X. Images of three representative moMΦ, one from each condition (untreated, IL-10, TGF-β) are displayed. Scale bar, 25 µm. (**b**) Changes in dimension and granularity of moMΦ were evaluated by flow cytometry. Forward scatter (FSC) and side scatter (SSC) data are presented as fold-change relative to untreated moMΦ. Mean data for quadruplicate biological replicates and standard error of the mean (SEM) are shown. Values of stimulated samples were compared to the untreated control using a one-way ANOVA followed by a Dunnett’s multiple comparison test.

Mφ stimulated with IL-10 and TGF-β were assessed by confocal microscopy and flow cytometry (Figure 1). moMΦ presented with a spherical shape with short hairy protrusions on their surface, as previously observed [20], and stimulation with IL-10 or TGF-β did not affect their morphology, dimension, or granularity (Figure 1).

**Figure 2:**
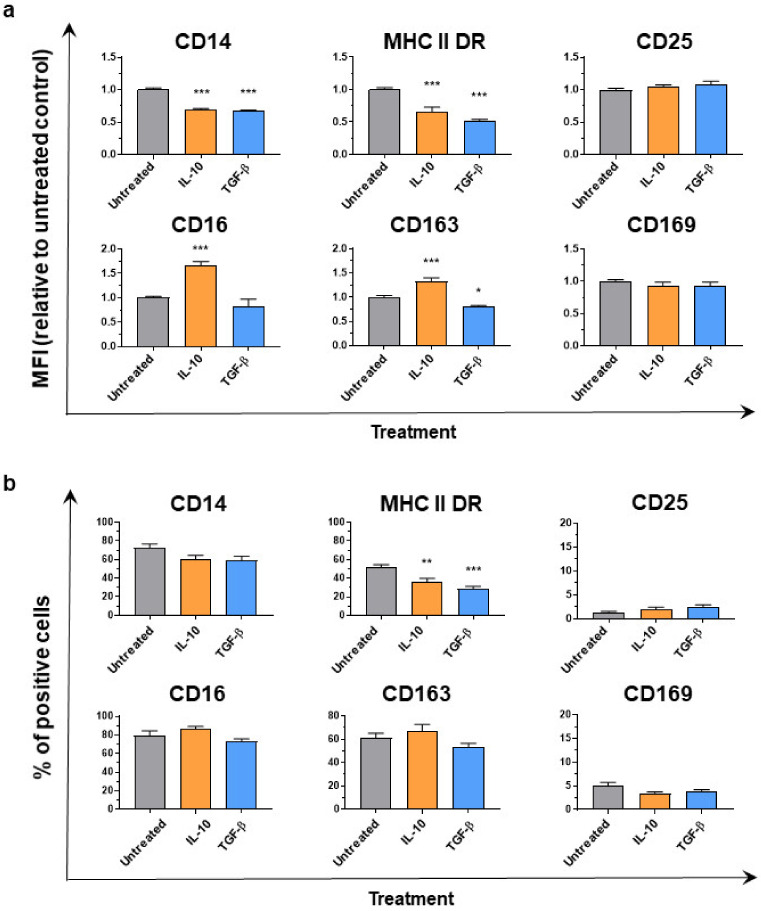
Effect of IL-10 or TGF-β on moMΦ surface marker expressions. 24 h post-stimulation with either IL-10 or TGF-β, surface expression of CD14, MHC II DR, CD25, CD16, CD163, and CD169 were assessed by flow cytometry. In Panel (**a**), mean fluorescence intensity (MFI) data are presented as fold-change relative to the untreated condition (moMΦ). In Panel (**b**), percentages of positive cells are shown. For both Panels b and c, mean data and SEM from four independent experiment using different animals are displayed. Values of stimulated samples were compared to the corresponding un-treated control (moMΦ) using a one-way ANOVA followed by a Dunnett’s multiple comparison test. Asterisks indicate statistically significant differences: *** *p* < 0.001, ** *p* < 0.01, * *p* < 0.05.

Cytokine treatment modulated the expression of surface markers on Mφ (Figure 2a). Both cytokines induced CD14 and MHC II DR down-regulation, with a statistically significantly (*p* < 0.001) reduced mean fluorescence intensity (MFI) observed in stimulated compared to untreated moMΦ. Stimulation with either cytokine also resulted in reduction of percentages of MHC II DR^+^ cells (Figure 2b). Stimulation with IL-10, but not TGF-β, induced up-regulation of both CD16 and CD163 on moMΦ (Figure 2a), but no differences were observed between untreated and IL-10 stimulated moMΦ in terms of percentages of CD16^+^ or CD163^+^ cells (Figure 2b). IL-10 and TGF-β did not alter CD25 and CD169 expression on moMΦ, either in terms of MFI or percentages of cells expressing either of these markers (Figure 2).

### 3.2. Induction of Cytokine Gene Expression and Release from Porcine moMΦ in Response to IL-10 or TGF-β Stimulation

**Figure 3:**
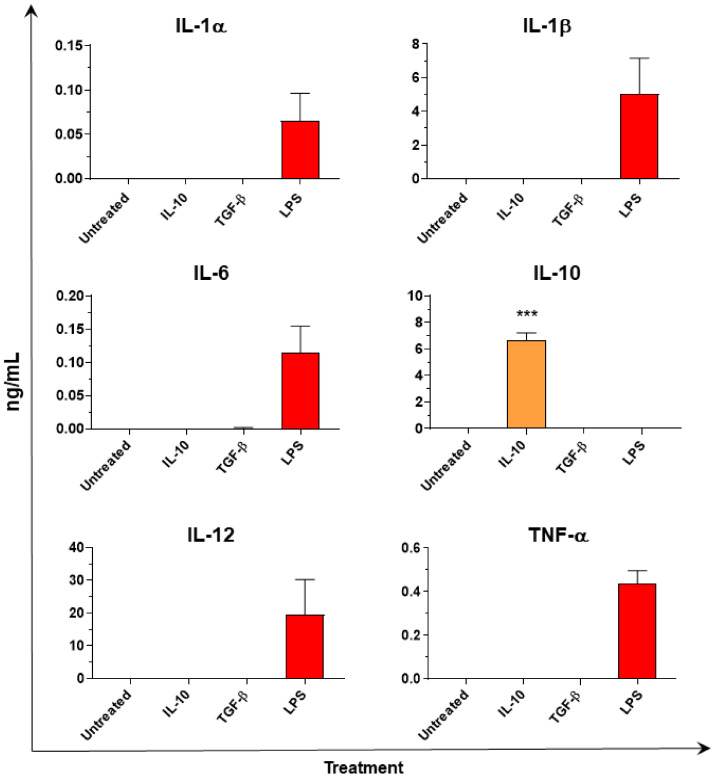
Cytokine release of moMΦ stimulated with IL-10 or TGF-β. At 24 h post stimulation with either IL-10 or TGF-β, levels of IL-1α, IL-1β, IL-6, IL-10, IL-12, TNF-α in culture supernatants were quantified using a multiplex ELISA. Lipopolysaccharide—LPS (1 mg/mL) was used as positive control for IL-1α, IL-1β, IL-6, IL-12, and TNF-α. Mean data and SEM from three independent experiment using different animals are shown. Values for IL-10 or TGF-β stimulated samples were compared to the corresponding untreated control (moMΦ) using a one-way ANOVA followed by a Bonferroni’s multiple comparison test. Asterisks indicate statistically significant differences: *** *p* < 0.001.

Multiplex ELISA analysis of moMΦ culture supernatants revealed no production of either pro-inflammatory cytokines (IL-1β, IL-6, TNF-α) or IL-12 in response to both IL-10 and TGF-β (Figure 3). IL-10 was detected in the supernatants of IL-10 stimulated moMΦ, however the amount detected 24 h post-stimulation (6.66 + 1.36 ng/mL) was below the amount added to culture media at time 0 (20 ng/mL), suggesting that no de-novo synthesis of IL-10 had occurred (Figure 3).

**Figure 4:**
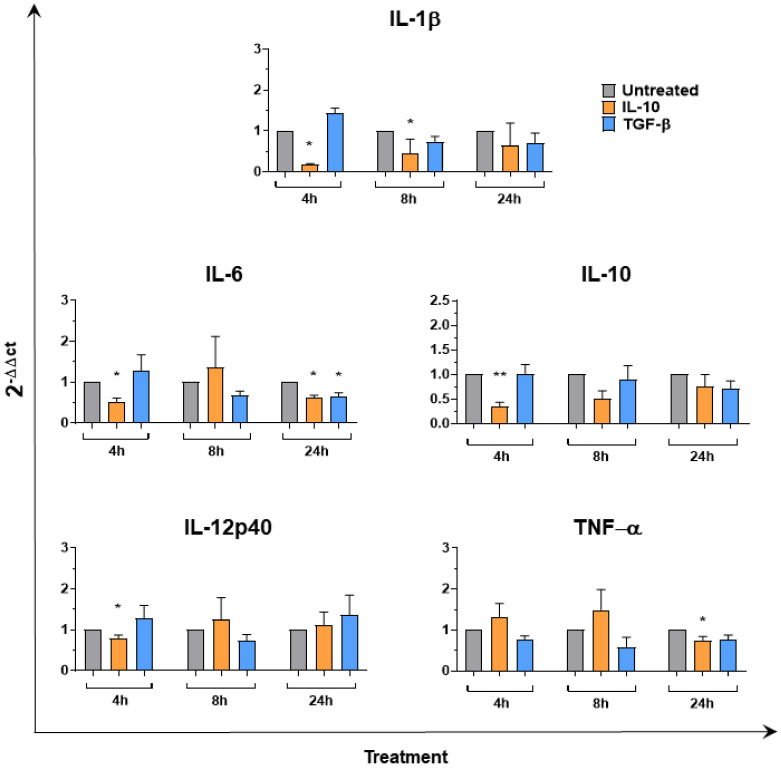
Cytokine gene expression in moMΦ following stimulation with IL-10 or TGF-β. moMΦ were left untreated or stimulated with IL-10 or TGF-β. At 4, 8, and 24 h post-stimulation, gene expression levels of IL-1β, IL-6, IL-10, IL-12, TNF-α were determined using qPCR. At each time point, data were normalized on the values of un-treated control and expressed as 2−ΔΔCt, with ΔCt = Ct (target gene) − Ct (reference gene), and ΔΔCt = ΔCt (stimulated samples) − ΔCt (un-treated sample, moMF). Mean data and SEM from five independent experiments using different animals are shown. For each time point, values of IL-10 or TGF-β stimulated samples were compared to the corresponding untreated control (moMΦ) using a Kruskal–Wallis multiple comparison test. Asterisks indicate statistically significant differences: ** *p* < 0.01, * *p* < 0.05.

To further explore the effects of IL-10 and TGF-β on Mφ, gene expression of several pro-inflammatory cytokines (IL-1β, IL-6, TNF-α), IL-12p40 and IL-10 were investigated at 4, 8, and 24 h post-simulation. RT-qPCR data revealed that neither IL-10 nor TGF-β induced enhanced expression of any of the tested cytokines. On the contrary, both recombinant cytokines reduced IL-6 gene expression 24 h post-stimulation (Figure 4). Treatment with IL-10 also induced a statistically significant down-regulation of IL-1β (4 and 8 h), IL-12p40 (4 h), TNF-α (24 h) expression (Figure 4). In humans and mice, increased IL-10 gene expression is a hallmark of M2c polarization [28], but we observed that neither IL-10 nor TGF-β enhanced IL-10 expression in porcine moMΦ. On the contrary, stimulation with recombinant IL-10 resulted in statistically significant down-regulated IL-10 gene expression 4 h post-stimulation (Figure 4).

### 3.3. IL-10 and TGF-β Inhibit moMΦ Responses to Stimulation with TLR4 and TLR2 Agonists

**Figure 5:**
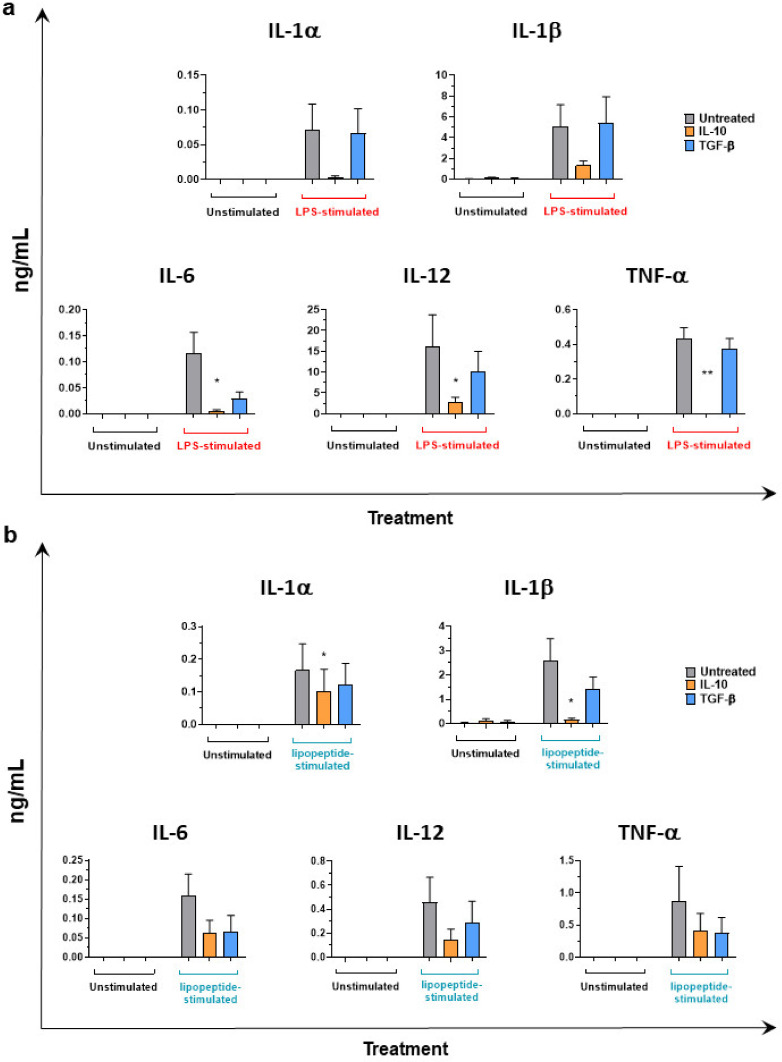
Impact of IL-10 or TGF-β on macrophage response to LPS stimulation. moMΦ were left untreated or stimulated with IL-10 and TGF-β. After 24 h, culture supernatants were replaced with fresh media and cells were left untreated or activated using LPS (1 mg/mL) (**a**) or pam2cys lipopeptide (100 ng/mL) (**b**). 24 h later, the amount of IL-1α, IL-1β, IL-6, IL-12, TNF-α in culture supernatants were evaluated using a multiplex ELISA. For both panels, the mean data and SEM from three independent experiments utilizing different blood donor pigs are shown. Values of IL-10 or TGF-β stimulated samples were compared to the corresponding un-treated control (moMΦ) using a Kruskal–Wallis multiple comparison test. Asterisks indicate statistically significant differences: ** *p* < 0.01, * *p* < 0.05.

The immunosuppressive ability of these two cytokines was next assessed. moMΦ were first stimulated for 24 h with Il-10 or TGF-β or left untreated, then supernatants were removed, and cells were activated with a TLR4 (LPS) or a TLR2 (Pam2Cys lipopeptide) agonist. Then, 24 h later, culture supernatants were collected, and multiplex ELISA analysis performed. IL-10 stimulation resulted in a reduced ability of moMΦ to release IL-1α, IL-1β, IL-6, IL-12, and TNF-α in response to stimulation with either LPS (Figure 5a) or Pam2Cys lipopeptide (Figure 5b).

Stimulation with 20 ng/mL of TGF-β did not statistically significantly impair the ability of moMΦ to release IL-1α, IL-1β, IL-6, IL-12, and TNF-α in response to either LPS or Pam2Cys lipopeptide, although a decreasing trend was observed for IL-6 and IL-12 in response to both stressors and for TNF-α in response to the TLR2 agonist (Figure 5).

### 3.4. CD14, TLR4, Arg-1 Expression in Macrophages Stimulated with IL-10 or TGF-β

**Figure 6:**
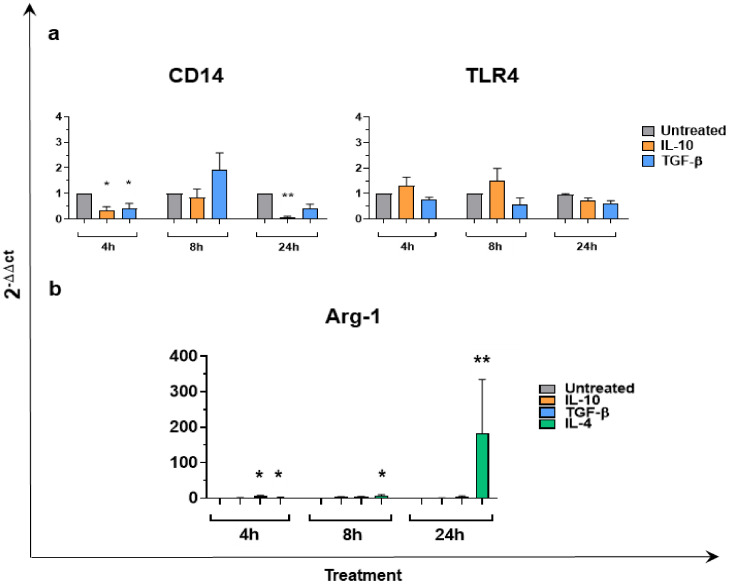
CD14, TLR4, Arg-1 gene expression in moMΦ following stimulation with IL-10 or TGF-β. moMΦ were left untreated or stimulated with IL-10 or TGF-bβ. At 4, 8, and 24 h post stimulation, gene expression levels of CD14, TLR4 Panel (**a**), Arg-1 Panel (**b**) were determined using RT-qPCR. For Arg-1 gene expression Panel (b), IL-4 stimulation was included as a positive control. For both panels, at each time-point, data were normalized on the values of untreated controls and expressed as 2−ΔΔCt, with ΔCt = Ct (target gene) − Ct (reference gene), and ΔΔCt = ΔCt (stimulated samples) − ΔCt (untreated sample). Mean data and SEM from five independent experiment using different blood donor pigs are shown. For each time point, values of stimulated samples were compared to the corresponding untreated control (moMΦ) using a Kruskal–Wallis multiple comparison test. Asterisks indicate statistically significant differences: ** *p* < 0.01, * *p* < 0.05.

We next investigated the ability of IL-10 and TGF-β to modulate CD14 and TLR4 (two molecules involved in bacterial components recognition) gene expression over time (4, 8, and 24 h post-simulation). As described in Figure 6a, both cytokines reduced CD14 gene expression, with statistical significance at 4 h (for both IL-10 and TGF-β) and at 24 h (only for IL-10). On the contrary, TLR4 expression was not altered by the two cytokines (Figure 6a). Finally, the ability of IL-10 or TGF-β to induce Arg-1 expression was assessed. Induction of this enzyme is considered a hallmark of M2a polarization in many species, including in swine [9]. IL-4 was used as positive control and as expected, a marked enhancement of Arg-1 expression was observed over time (Figure 6b). Neither IL-10 nor TGF-β triggered Arg-1 induction, except for a modest but statistically significant enhancement observed in TGF-β treated moMΦ at 4 h post-stimulation. Nevertheless, enhanced expression of Arg-1 was detected in only four out of five tested subjects.

## 4. Discussion

Mφ are phagocytic cells which play a central role in innate immune response to both infectious and non-infectious stressors [3]. Diversity and plasticity are properties of this heterogeneous family, which allows modifications to their phenotype and functions in response to changes in the surrounding microenvironment [29]. In a spectrum of polarizing states, M1 and M2 represent two antithetic extremes [29]. Stimulation of human or murine Mφ with either IL-10 or TGF-β or glucocorticoids polarize Mφ toward an M2c state, which promotes anti-inflammatory activities and phagocytosis of apoptotic cells [4,28]. However, limited information is available on the existence of an M2c phenotype in pigs. Two studies focused on the effects of IL-10 and dexamethasone on porcine monocyte and moMφ phenotypes and susceptibility to PRRSV [7,30]. In our study, for the first time, the phenotypical and functional impact of IL-10 and TGF-β on porcine Mφ was investigated in detail.

Phenotype was first investigated. Our data revealed that stimulation with either IL-10 or TGF-β reduced MHC Class II expression on Mφ, in accordance with what was reported in other species [2]. Stimulation with these cytokines also down-regulated surface expression of CD14, supporting the immunosuppressive effect of these two cytokines in swine. Their anti-inflammatory features were reflected also by the RT-qPCR data, which revealed that both cytokines reduced expression of pro-inflammatory cytokines (IL-6 and TNF-α) and CD14. Differences between the effects of IL-10 and TGF-β were also observed: stimulation with IL-10, but not TGF-β, resulted in enhanced expression of CD163 and CD16. M2 cells are characterized by high levels of scavenger receptors, like CD163 [28,31], and previous studies described that IL-10 induced CD163 upregulation on both porcine monocytes and Mφ [7,30]. Similar findings were described in humans, where IL-10 stimulation enhanced both CD16 and CD163 expression on Mφ [32], and TGF-β decreased CD163 expression [33]. Dissimilarities between the effects of the two cytokines were also revealed also by the RT-qPCR and cytokine ELISA data. Stimulation with IL-10, but not TGF-β, resulted in marked reduction of IL-1β and IL-12p40 gene expression early post-stimulation and drastically impaired the ability of moMΦ to release IL-12 and pro-inflammatory cytokines (IL-1α, IL-1β, IL-6, TNF-α) in response to TLR2 and TLR4 agonists. Interestingly, stimulation with TGF-β did not statistically significantly impair the ability of moMΦ to respond to these stimuli, although a clear trend was observed for IL-6 and IL-12 responses. Overall, our results highlight differences in the polarizing effects of these two cytokines, suggesting that it would be more appropriate to use a nomenclature linked to the activator(s) used, such as M(IL-10), M(TGF-β), as suggested by Murray et al. [6].

Some peculiarities in responses of porcine Mφ were also observed. Enhanced IL-10 expression and release is regarded as a hallmark of M2c polarization in both humans and mice [28,31], but in our study, we observed that neither IL-10 or TGF-β triggered enhanced expression or release of this cytokine. In addition, a reduced IL-10 gene expression was observed in porcine moMΦ 4h post-stimulation with IL-10. Similar findings were observed in porcine M2a; with no release of IL-10 by porcine Mφ in response to IL-4 stimulation [9,34], whereas in both humans and mice, exposure to IL-4 results in production of high levels of IL-10 and chemokines that promotes recruitment of Th2 cells [5,22]. Future studies should investigate this particularity of pigs, which should be also considered in translational studies.

We finally evaluated IL-10 and TGF-β ability to induce Arg-1 gene expression. Induction of Arg-1 is considered a hallmark of M2a polarization in humans, mice [35] and pigs [9], and we confirmed in this study enhanced expression of Arg-1 following IL-4 stimulation. Arg-1 catalyzes the hydrolysis of arginine to ornithine, resulting in increased polyamine synthesis; the latter playing an important role in tissue repair and remodeling [35]. Previous studies in rodents reported that both cytokines increased arginase activity in Mφ [36,37] and IL-10 further enhanced Arg-1 expression in IL-4 activated M2a [38]. On the contrary, Arg-1 is not an M2c marker in human [28]. In this study, we observed that in pigs, IL-10 did not induce expression of this enzyme, instead a modest but statistically significant enhancement of Arg-1 expression was observed in moMΦ at 4 h post-stimulation with TGF-β. Nevertheless, no differences between treated and untreated cells were detected at later time points. Many differences between species exist in terms of arginine pathways in Mφ [39]; this pathway plays an important role in immunopathology; thus, these dissimilarities should be better characterized in future studies.

## 5. Conclusions

This study has provided a detailed characterization of the polarizing effect of IL-10 and TGF-β on porcine moMΦ. Both cytokines induced CD14 and MHC II DR down-regulation, as well as reductions in IL-6 and TNF-α gene expression reflecting their immunosuppressive ability. Phenotypic and functional differences between moMΦ (IL-10) and moMΦ (TGF-β) were also observed: stimulation with IL-10, but not TGF-β, resulted in enhanced CD16 and CD163 upregulation, down-regulated expression of IL-1β and IL-12p40 and induced a stronger impairment of moMΦ ability to respond to TLR2 or TLR4 agonists. We also highlighted a peculiarity of swine MΦ: neither IL-10 or TGF-β stimulation enhanced IL-10 expression or release in porcine moMΦ. The information generated by this study helps further characterize the complex and heterogeneous Mφ family in pigs and to highlights differences between species, that should be considered in translational studies.

## Data Availability

The data presented in this study are available on request from the corresponding author.
